# Correction to: Topical Low Dose Preservative-Free Hydrocortisone Reduces Signs and Symptoms in Patients with Chronic Dry Eye: A Randomized Clinical Trial

**DOI:** 10.1007/s12325-019-01190-3

**Published:** 2019-12-10

**Authors:** Martin Kallab, Stephan Szegedi, Nikolaus Hommer, Hannes Stegmann, Semira Kaya, René M. Werkmeister, Doreen Schmidl, Leopold Schmetterer, Gerhard Garhöfer

**Affiliations:** 1 Department of Clinical Pharmacology, Medical University of Vienna, Vienna, Austria; 2 Center for Medical Physics and Biomedical Engineering, Medical University of Vienna, Vienna, Austria; 3 Department of Ophthalmology, Hietzing Hospital, Vienna, Austria; 4 Singapore Eye Research Institute, Singapore, Singapore; 5 Nanyang Technological University, Singapore, Singapore; 6 Ophthalmology and Visual Sciences Academic Clinical Program, Duke-NUS Medical School, Singapore, Singapore

## Correction to: Adv Ther 10.1007/s12325-019-01137-8

The article “Topical Low Dose Preservative-Free Hydrocortisone Reduces Signs and Symptoms in Patients with Chronic Dry Eye: A Randomized Clinical Trial”, written by Martin Kallab, Stephan Szegedi, Nikolaus Hommer, Hannes Stegmann, Semira Kaya, René M. Werkmeister, Doreen Schmidl, Leopold Schmetterer, Gerhard Garhöfer was originally published electronically on the publisher’s internet portal (currently SpringerLink) on November 19, 2019 without open access.

The copyright of the article changed to © The Author(s) 2019 and the article is forthwith distributed under the terms of the Creative Commons Attribution-NonCommercial 4.0 International License (http://creativecommons.org/licenses/by-nc/4.0/), which permits any non-commercial use, distribution, and reproduction in any medium, provided you give appropriate credit to the original author(s) and the source, provide a link to the Creative Commons license, and Indicate if changes were made.

The original article has been updated.

